# RISK: a next-generation tool for biological network annotation and visualization

**DOI:** 10.1093/bioinformatics/btaf669

**Published:** 2025-12-19

**Authors:** Ira Horecka, Hannes Röst

**Affiliations:** Terrence Donnelly Centre for Cellular & Biomolecular Research, University of Toronto, Toronto, ON M5S 3E1, Canada; Department of Molecular Genetics, University of Toronto, Toronto, ON M5S 1A8, Canada; Terrence Donnelly Centre for Cellular & Biomolecular Research, University of Toronto, Toronto, ON M5S 3E1, Canada; Department of Molecular Genetics, University of Toronto, Toronto, ON M5S 1A8, Canada; Department of Computer Science, University of Toronto, Toronto, ON M5S 2E4, Canada

## Abstract

**Summary:**

Analyzing biological networks demands scalable annotation tools, yet existing methods fall short in clustering power, statistical flexibility, and broad data compatibility. We introduce Regional Inference of Significant Kinships (RISK), a next-generation tool that overcomes these challenges by integrating community detection algorithms, rigorous overrepresentation analysis, and a modular architecture that supports diverse network types. RISK identifies biologically coherent relationships within networks and generates publication-ready visualizations, as demonstrated by its ability to resolve compact functional modules in *Saccharomyces cerevisiae* protein–protein interaction and genetic interaction networks. Its application to a high-energy physics citation network reveals structured relationships among research subfields, highlighting its versatility beyond biological systems. As biological and interdisciplinary networks increase in size and complexity, RISK’s scalability and adaptability make it a powerful solution for modern network analysis.

**Availability and implementation:**

RISK is compatible with Python 3.8 or later, supports all major operating systems, and can be installed via pip. The software is open source under the GPLv3 license on GitHub (https://github.com/riskportal/risk) and archived on Zenodo (https://doi.org/10.5281/zenodo.17257418). Documentation and a step-by-step Jupyter notebook tutorial are available at https://github.com/riskportal/risk-docs.

## 1 Introduction

Biological networks map interactions that govern cellular processes and disease mechanisms (Barabási and Oltvai 2004, Vidal *et al.* 2011). These networks define regulatory pathways and therapeutic targets, yet their increasing size and complexity make interpretation challenging. Scalable annotation tools are essential for uncovering functional relationships in such datasets.

We present Regional Inference of Significant Kinships (RISK), a next-generation tool for annotating and visualizing biological networks. RISK’s modular architecture supports diverse data formats and integrates seamlessly into bioinformatics workflows. It combines clustering algorithms, rigorous statistical analysis, and a visualization framework to enhance interpretation of biological networks. RISK identifies structured network regions using algorithms such as Louvain, Leiden, and Markov Clustering. It includes a suite of statistical tests—ranging from fast analytical approximations to rigorous permutation testing—to evaluate significantly overrepresented biological processes within these clusters and visualizes these relationships as high-resolution layouts that drive network exploration.

Several methods exist for annotating biological networks (Maere *et al.* 2005, Yu *et al.* 2012, McLean *et al.* 2023). Spatial Analysis of Functional Enrichment (SAFE; Baryshnikova 2016) is a widely adopted framework that uses predefined spatial neighborhoods—such as shortest-path and Euclidean distances—to integrate network topology with functional enrichment. Although effective for localized annotation, its reliance on predefined spatial heuristics limits its adaptability to large networks with complex modular structures.

RISK extends SAFE’s approach by detecting biologically meaningful clusters without such constraints. RISK scales efficiently to networks exceeding hundreds of thousands of edges on standard hardware, handling networks an order of magnitude larger than SAFE. Its scalable modular architecture, robust statistical framework, and high-resolution visualization make RISK well-suited for large-scale network analysis.

To evaluate RISK’s performance, we compared it with SAFE on *Saccharomyces cerevisiae* protein–protein interaction (PPI; Michaelis *et al.* 2023) and genetic interaction (GI; Costanzo *et al.* 2016) networks. RISK identified more compact clusters with improved silhouette scores, uncovering additional modules that reveal the organization of cellular processes. To demonstrate RISK’s versatility beyond biology, we applied it to a high-energy physics citation network (Gehrke *et al.* 2003, Leskovec *et al.* 2005, Leskovec and Krevl 2014), where it resolved structured research subfields.

These results establish RISK as a scalable solution for robust network analysis. RISK integrates flexible clustering, a rigorous statistical framework, and a modular architecture to enhance functional discovery across diverse datasets, positioning it as a powerful tool in modern network science. With broad file format support, intuitive software design, and thorough documentation, RISK can be immediately adopted into bioinformatics workflows.

## 2 Methods

### 2.1 Software design and functionality

**Figure 1.**
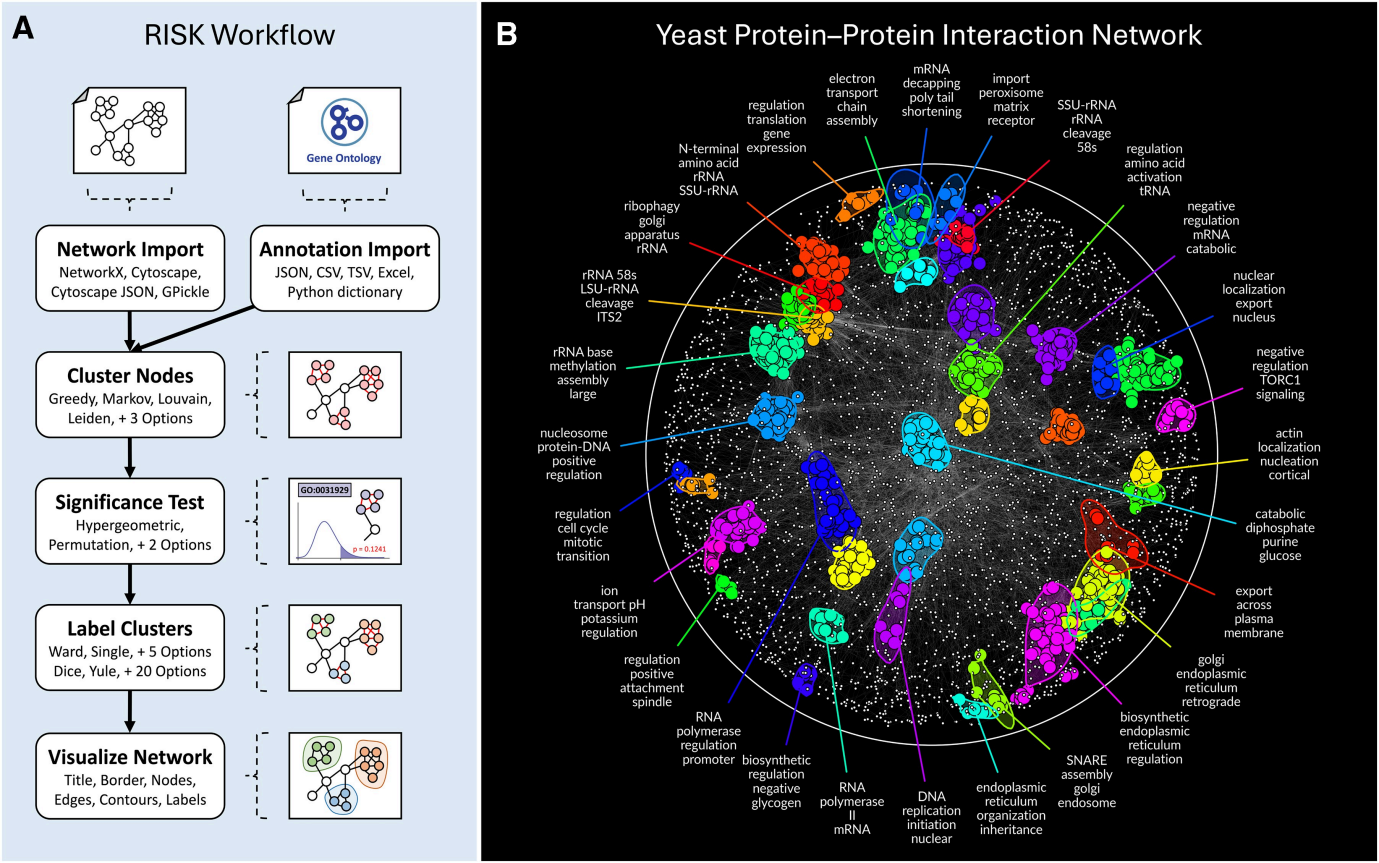
RISK workflow overview and analysis of the *Saccharomyces cerevisiae* protein–protein interaction (PPI) network. (A) Schematic overview of the RISK workflow, summarizing the process from network import and annotation through clustering (e.g. Louvain, Markov Clustering), statistical significance testing (including permutation and hypergeometric), and visualization. RISK’s modular architecture supports multiple input formats [e.g. Cytoscape (Shannon *et al.* 2003), JSON, CSV] and offers configurable parameters for flexible network exploration. (B) RISK analysis of the yeast PPI network (3839 nodes, 30 955 edges; Michaelis *et al.* 2023). Clusters are color-coded to represent key cellular processes—as defined by Gene Ontology Biological Process (GO BP; Ashburner *et al.* 2000)—including ribosomal assembly, mitochondrial organization, and RNA polymerase activity.

RISK integrates seamlessly into existing workflows (Fig. 1A). It imports networks in Cytoscape (Shannon *et al.* 2003), Cytoscape JSON, GPickle, and NetworkX formats. It also accepts user-provided annotation data—commonly sourced from established resources such as Gene Ontology (Ashburner *et al.* 2000), CORUM (Tsitsiridis *et al.* 2023), or KEGG (Kanehisa *et al.* 2023)—formatted as term–to–gene membership tables in JSON, CSV, TSV, Excel, and Python dictionaries.

RISK’s clustering module supports multiple clustering algorithms, including Greedy Modularity (Newman 2004), Label Propagation (Raghavan *et al.* 2007), Leiden (Traag *et al.* 2019), Louvain (Blondel *et al.* 2008), Markov Clustering (Van Dongen 2008), Spinglass (Reichardt and Bornholdt 2006), and Walktrap (Pons and Latapy 2005). Louvain and Leiden offer efficient clustering for large networks, whereas Markov Clustering and Walktrap offer better performance for compact modules in smaller networks. Users can define clustering parameters to adjust resolution according to node density and connectivity.

RISK’s statistical module assesses significance by testing for the overrepresentation of biological processes within network clusters using permutation, hypergeometric, chi-squared, and binomial. These tests range from fast approximations—such as chi-squared and binomial—to more computationally intensive but rigorous approaches—such as permutation—allowing users to balance efficiency and statistical robustness.

RISK’s visualization module provides precise control over network representation. Users can adjust node size, color, and edge weight to emphasize biologically significant regions, while contour overlays improve network interpretation by outlining functional modules. RISK exports figures in various formats, including SVG, PNG, and PDF, ensuring high-resolution output for exploratory analysis and publication.

RISK demonstrates computational efficiency across a suite of statistical tests, maintaining low execution times and efficient memory usage on standard hardware. Its modular architecture and statistical flexibility enable generalization across diverse datasets, providing a scalable framework for network annotation and visualization. RISK is cross-platform, runs on Python 3.8 or later, and is easily installable via pip: pip install risk-network.

### 2.2 Clustering performance, hypothesis generation, and scalability

We evaluated clustering quality and hypothesis generation by applying RISK—using the Louvain algorithm (Blondel *et al.* 2008)—and SAFE—using its published shortest-path neighborhood algorithm (Baryshnikova 2016)—to the yeast PPI network (3839 nodes, 30 955 edges; Michaelis *et al.* 2023) and the yeast GI network (3641 nodes, 23 562 edges; Costanzo *et al.* 2016), which was originally analyzed with SAFE. Only clusters with significant Gene Ontology Biological Process (GO BP; Ashburner *et al.* 2000) overrepresentation (*P* < .01, permutation test) were retained for visualization, except for the yeast GI network, where SAFE was evaluated at *P* < .05 to match its original analysis. Networks were visualized using a spring-embedded layout (Fruchterman–Reingold algorithm; Fruchterman and Reingold 1991), unless otherwise specified.

We defined clustering quality as compactness (mean pairwise Euclidean distance between nodes within clusters), separation (minimum distance between cluster centroids), and silhouette scores (Rousseeuw 1987). A Mann–Whitney U test assessed whether RISK and SAFE produced significantly different distributions of compactness and separation. Hypothesis quality was evaluated using GO BP overrepresentation analysis with the hypergeometric test.

To evaluate computational scalability, we benchmarked RISK and SAFE on simulated scale-free networks (Barabási and Albert 1999) with twice as many edges as nodes. Execution time and memory usage were measured across statistical tests to assess scalability on large networks.

### 2.3 Availability and licensing

RISK is open source under the GPLv3 license on GitHub (https://github.com/riskportal/risk). Documentation and a step-by-step Jupyter notebook tutorial are available at https://github.com/riskportal/risk-docs.

## 3 Results

### 3.1 Resolving functional modules in yeast interaction networks

We applied RISK to a *Saccharomyces cerevisiae* protein–protein interaction (PPI) network reported in Michaelis *et al.* (2023), which includes 3839 proteins and 30 955 interactions (Fig. 1B). RISK identified compact clusters overrepresented in Gene Ontology Biological Process (GO BP) terms, as defined by Ashburner *et al.* (2000). These functional modules reveal the organization of cellular processes, including ribosomal assembly, mitochondrial organization, and RNA polymerase activity. In the yeast genetic interaction (GI) network (3641 nodes, 23 562 edges; Costanzo *et al.* 2016, Fig. 1, available as supplementary data at *Bioinformatics* online), RISK identified modules associated with cell cycle regulation, vesicle transport, and DNA replication, demonstrating RISK’s ability to resolve biological organization across network types.

Clustering reveals modular network structures and is an essential step in biological network analysis. To evaluate clustering performance, we compared RISK with SAFE across yeast interaction networks. The yeast PPI network served as a benchmark, while the yeast GI network—originally analyzed with SAFE—provided a direct comparison. In both cases, RISK produced tighter, more coherent clusters, as reflected by improved cluster compactness (yeast PPI network: *P* < .0001; yeast GI network: *P* < .0001; Figs 2 and 3, available as supplementary data at *Bioinformatics* online) and higher silhouette scores (yeast PPI network: 0.45 versus 0.40; yeast GI network: 0.41 versus 0.18).

RISK is effective in resolving functional modules. Contour overlays of RISK clusters (41) and SAFE clusters (22) on the yeast PPI network revealed distinct differences in GO BP overrepresentation (Fig. 4, available as supplementary data at *Bioinformatics* online). After excluding nodes shared between significant clusters, RISK retained 16 unique clusters while SAFE retained 1. RISK uniquely identified clusters for TOR signaling, stress granule assembly, and nuclear pore localization. GO BP overrepresentation tables for RISK and SAFE summarize the biological significance of these clusters (Figs 5 and 6, available as supplementary data at *Bioinformatics* online). These results highlight RISK’s effectiveness in resolving functional modules and enhancing biological discovery.

### 3.2 Application to interdisciplinary networks

To evaluate RISK’s versatility, we applied it to a high-energy physics citation network (20 147 nodes, 331 044 edges; Gehrke *et al.* 2003, Leskovec *et al.* 2005, Leskovec and Krevl 2014) representing citations between papers, and we visualized the network using the ForceAtlas2 layout algorithm (Jacomy *et al*. 2014). RISK identified clusters corresponding to research subfields, including quantum mechanics and particle physics (*P* < .001, permutation test; Fig. 7, available as supplementary data at *Bioinformatics* online). The analysis highlighted structural patterns, such as methodological overlaps between general relativity and cosmology. In contrast, SAFE failed to detect significant clusters (*P* < .01). Together, these findings demonstrate RISK’s versatility in identifying structured relationships in large networks beyond biology.

### 3.3 Computational performance and scalability

With large-scale biological networks now readily available—e.g. a human protein–protein interaction network with 21 557 proteins and 342 353 interactions (Leskovec and Krevl 2014, Agrawal *et al.* 2018)—efficient network analysis becomes paramount. To address this challenge, we benchmarked RISK and SAFE on simulated scale-free networks to evaluate computational efficiency and scalability. We generated networks with twice as many edges as nodes, reflecting the power-law distributions characteristic of real-world systems (Barabási and Albert 1999), and assigned simulated annotations (1000 terms) based on GO BP membership distributions to model biologically relevant conditions.

Benchmarking focused on the statistical tests—the most computationally intensive step in large-scale analyses. We evaluated RISK and SAFE using their most efficient methods on standard hardware. RISK’s chi-squared test scaled to networks with 500 000 edges (250 000 nodes), whereas SAFE’s hypergeometric method experienced memory limitations beyond 50 000 edges (Fig. 8, available as supplementary data at *Bioinformatics* online). RISK achieves this scalability by performing statistical tests using vectorized operations on sparse matrices, minimizing memory use, whereas SAFE relies on dense neighborhood expansions. These results demonstrate RISK’s scalability for large network analysis.

## 4 Conclusion

RISK combines advanced clustering, a robust statistical framework, and a modular architecture to reveal functional relationships in networks. It outperforms SAFE—the state-of-the-art network annotation tool—in biological discovery and computational performance. Its ability to identify underexplored modules makes hypothesis generation reproducible and scalable on large networks. By addressing the challenges of modern, large-scale datasets, RISK provides an efficient, adaptable solution for network analysis that drives discoveries across biological and interdisciplinary fields.

## Supplementary Material

btaf669_Supplementary_Data

## Data Availability

All data supporting this study are available in publicly accessible repositories. The RISK Documentation repository (https://github.com/riskportal/risk-docs) provides documentation and a step-by-step Jupyter notebook tutorial for using the RISK software with the *Saccharomyces cerevisiae* protein–protein interaction network. The RISK Publication repository (https://github.com/riskportal/risk-publication) includes processed datasets, analysis scripts, and workflows for generating all figures presented in this study. Please contact the corresponding author for further inquiries regarding the data or analysis methods.
