# Supplementary material for: RISK: a next-generation tool for biological network annotation and visualization
Source: Bioinformatics. 2025 Dec 19;42(1):btaf669. doi: 10.1093/bioinformatics/btaf669 (PMC12766918; doi:10.1093/bioinformatics/btaf669)
Supplement: btaf669_Supplementary_Data [file btaf669_supplementary_data.zip › Supplementary Figures - BIOINF-2025-0598.pdf]

Supplementary Figures

This document contains supplementary figures supporting the analyses presented in the manuscript, "RISK: a next-generation tool for biological network annotation and visualization." Each figure is accompanied by a legend describing the analysis performed and key observations.

- **Supplementary Figure S1**: RISK analysis of the *Saccharomyces cerevisiae* genetic interaction (GI) network (Costanzo *et al*., 2016), highlighting key cellular processes.
- **Supplementary Figure S2**: Comparative clustering analysis of RISK and SAFE on the yeast protein–protein interaction (PPI) network (Michaelis *et al*., 2023).
- **Supplementary Figure S3**: Comparative clustering analysis of RISK and SAFE on the yeast GI network.
- **Supplementary Figure S4**: Contours comparing RISK and SAFE clusters in the yeast PPI network, highlighting differences in biological overrepresentation patterns.
- **Supplementary Figure S5**: Overrepresented Gene Ontology Biological Process (GO BP; Ashburner *et al*., 2000) terms identified by RISK in the yeast PPI network.
- **Supplementary Figure S6**: Overrepresented GO BP terms identified by SAFE in the yeast PPI network.
- **Supplementary Figure S7**: RISK analysis of a high-energy physics citation network (Gehrke *et al*., 2003; Leskovec *et al*., 2005; Leskovec and Krevl, 2014), illustrating interdisciplinary applicability.
- **Supplementary Figure S8**: Benchmarking execution time and memory usage for RISK and SAFE across multiple statistical tests on simulated scale-free networks (Barabási and Albert, 1999).

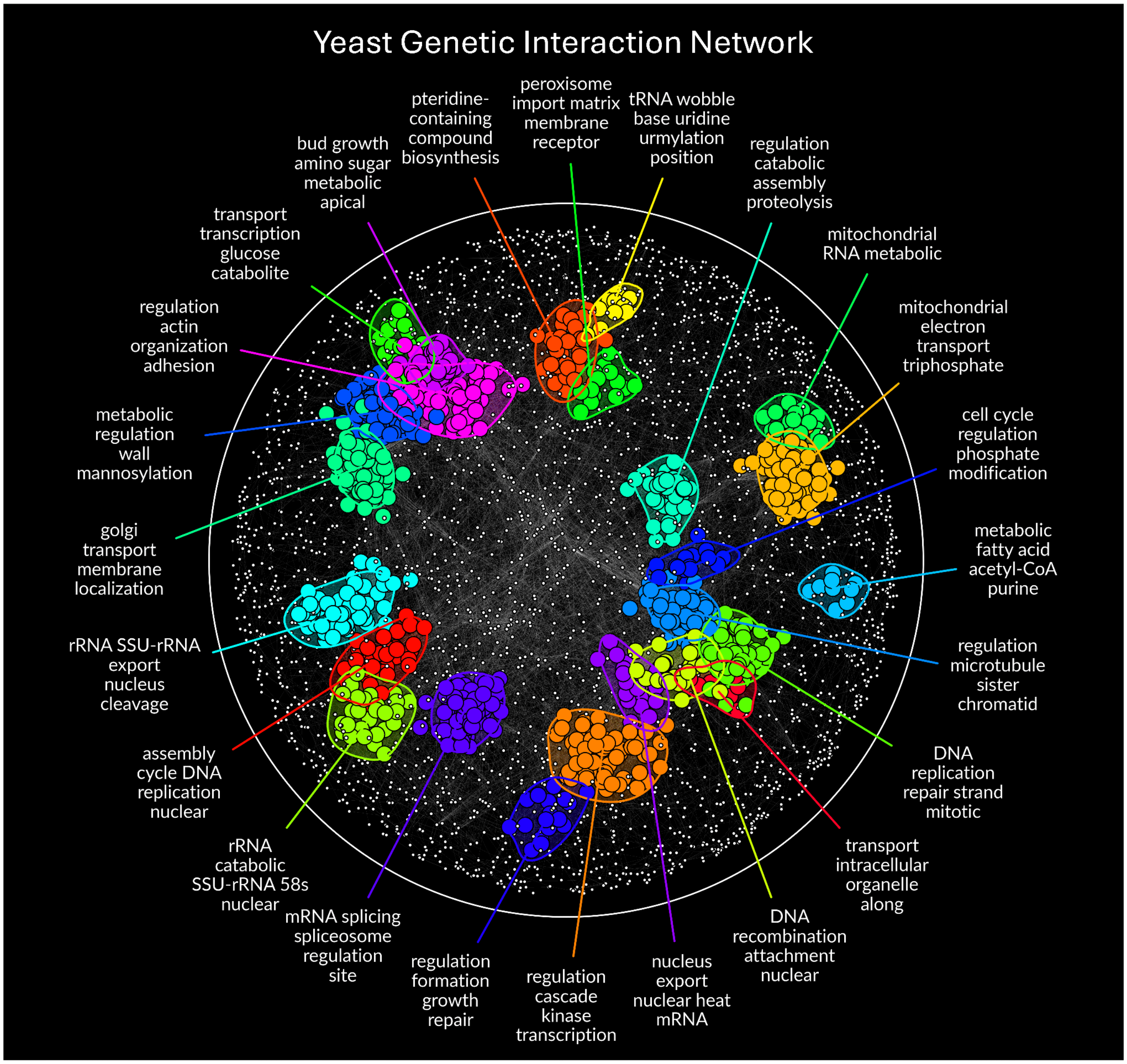

Supplementary Figure S1. RISK analysis of the *Saccharomyces cerevisiae* genetic interaction (GI) network (3,641 nodes, 23,562 edges; Costanzo *et al*., 2016). Gene Ontology Biological Process (GO BP) terms—as defined by Ashburner *et al*. (2000)—are color-coded to represent key cellular processes, including cell cycle regulation, vesicle transport, and DNA replication.

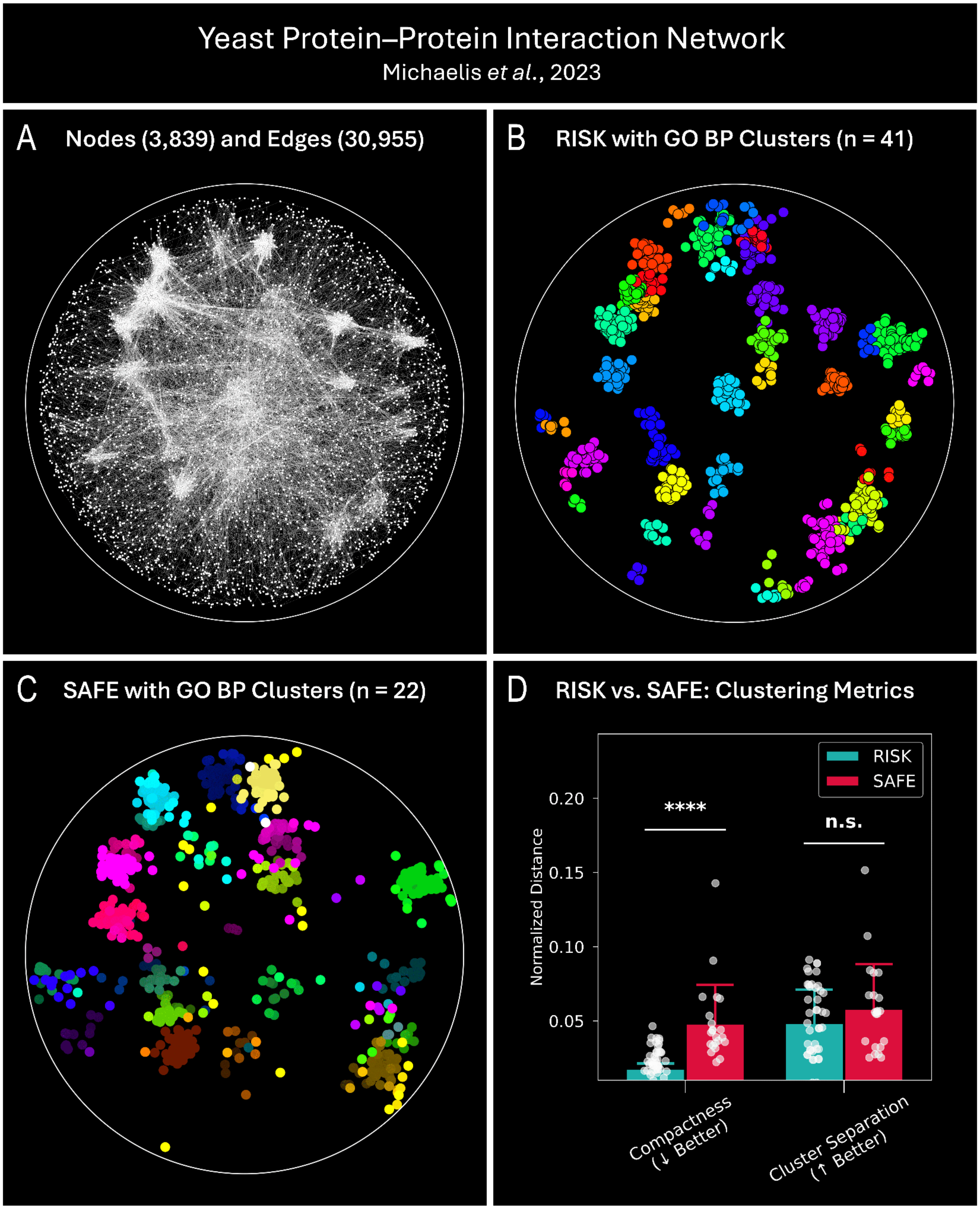

Yeast Protein–Protein Interaction Network
Michaelis et al., 2023
A Nodes (3,839) and Edges (30,955)
B RISK with GO BP Clusters (n = 41)
C SAFE with GO BP Clusters (n = 22)
D RISK vs. SAFE: Clustering Metrics
RISK
SAFE
0.20
0.15
0.10
0.05
Normalized Distance
****
n.s.
Compactness (↓ Better)
Cluster Separation (↑ Better)

Supplementary Figure S2. Comparative analysis of the yeast protein–protein interaction (PPI) network using RISK and SAFE. (**A**) The yeast PPI network with 3,839 nodes and 30,955 edges (Michaelis *et al*., 2023). (**B**) RISK identifies 41 GO BP clusters—as defined by Ashburner *et al*. (2000)—using the Louvain algorithm (Blondel *et al*., 2008). (**C**) SAFE identifies 22 GO BP clusters using its default visualization package and shortest-path algorithm (Baryshnikova, 2016). (**D**) Quantitative comparison of clustering metrics. RISK exhibits significantly improved cluster compactness ($p < 0.0001$) and higher silhouette scores (0.45 vs. 0.40). Statistical comparisons are performed using the Mann-Whitney U test.

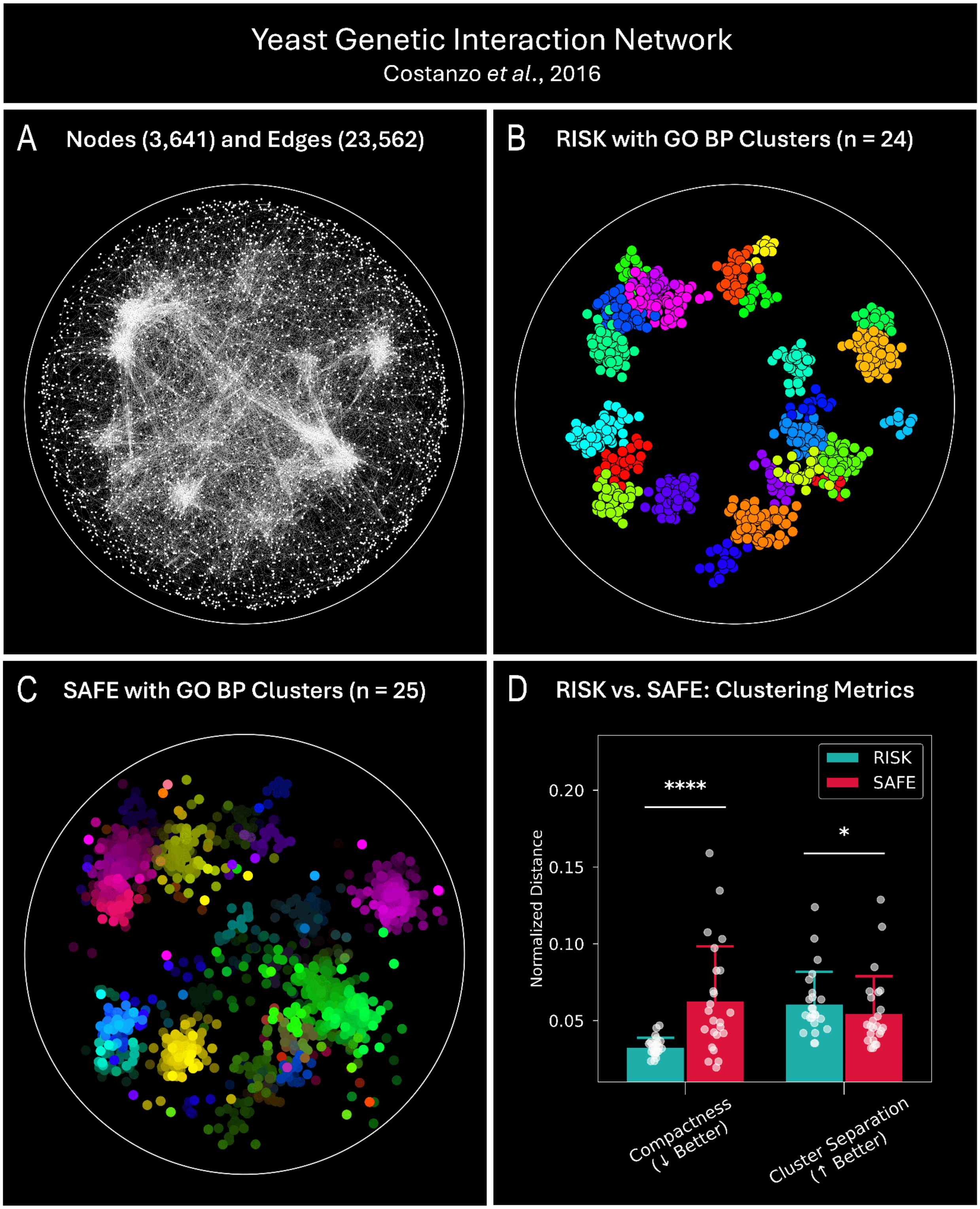
Yeast Genetic Interaction Network
Costanzo et al., 2016
A Nodes (3,641) and Edges (23,562)
B RISK with GO BP Clusters (n = 24)
C SAFE with GO BP Clusters (n = 25)
D RISK vs. SAFE: Clustering Metrics
RISK
SAFE
****
*
0.20
0.15
0.10
0.05
Normalized Distance
Compactness (↓ Better)
Cluster Separation (↑ Better)

Supplementary Figure S3. Comparative analysis of the yeast GI network using RISK and SAFE. (**A**) The yeast GI network with 3,641 nodes and 23,562 edges (Costanzo *et al*., 2016). The network was used without modification to ensure a fair comparison with the original SAFE analysis. (**B**) RISK identifies 24 GO BP clusters—as defined by Ashburner *et al*. (2000)—using the Louvain algorithm (Blondel *et al*., 2008). (**C**) SAFE identifies 25 GO BP clusters using its default visualization package and shortest-path algorithm (Baryshnikova, 2016). (**D**) Quantitative comparison of clustering metrics. RISK exhibits significantly improved cluster compactness ($p < 0.0001$), greater cluster separation ($p < 0.05$), and higher silhouette scores (0.41 vs. 0.18). Statistical comparisons are performed using the Mann-Whitney U test.

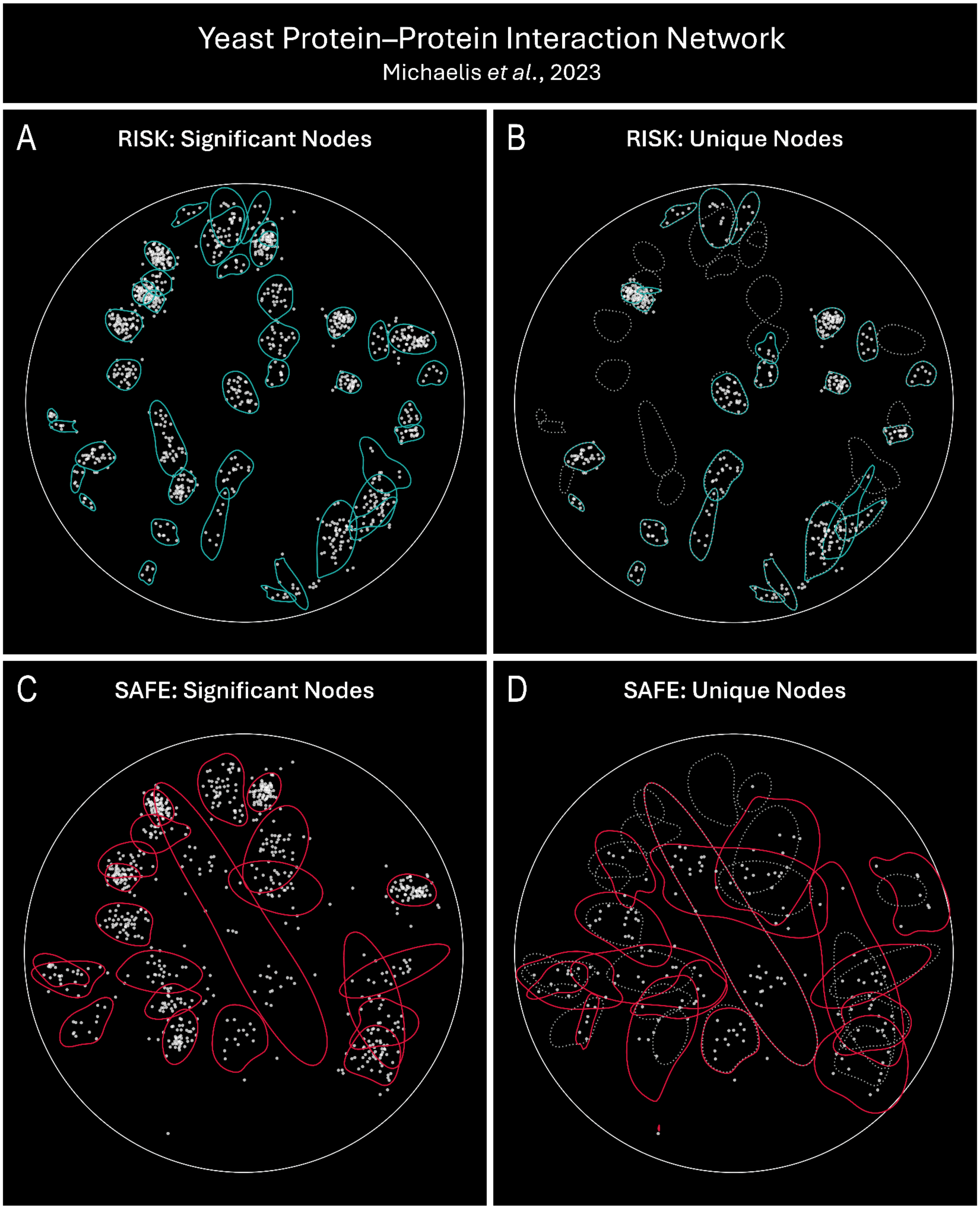
Yeast Protein–Protein Interaction Network
Michaelis et al., 2023
A
RISK: Significant Nodes
B
RISK: Unique Nodes
C
SAFE: Significant Nodes
D
SAFE: Unique Nodes

Supplementary Figure S4. Comparative analysis of RISK and SAFE in identifying biologically significant clusters in the yeast PPI network (Michaelis *et al*., 2023). (**A**) RISK identifies 41 GO BP clusters—as defined by Ashburner *et al*. (2000)—using the Louvain algorithm (Blondel *et al*., 2008), with contour overlays outlining functional groupings of biologically significant nodes. (**B**) After excluding biologically significant nodes shared with SAFE, RISK identifies 16 cohesive clusters. (**C**) SAFE identifies 22 GO BP clusters using the shortest-path algorithm (Baryshnikova, 2016). (**D**) After excluding biologically significant nodes shared with RISK, SAFE identifies a single diffuse cluster.

Yeast Protein–Protein Interaction Network

Michaelis *et al.*, 2023

RISK with GO BP Clusters (n = 41)

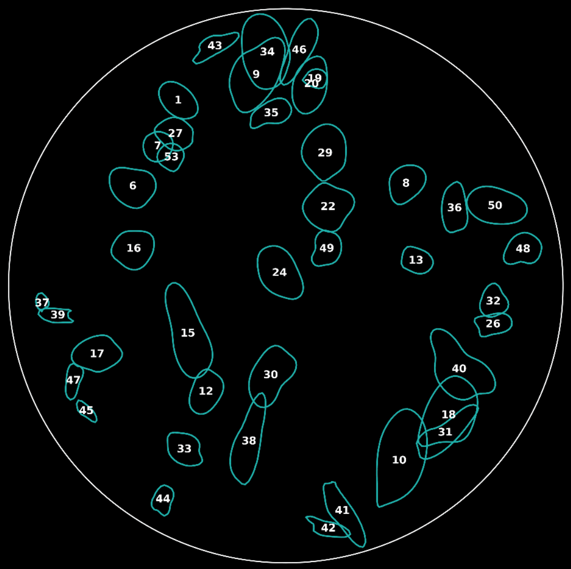

Cluster ID	Term	P-Value	FDR
1	ribosomal small subunit assembly	1.94e-21	3.54e-18
6	ribosomal large subunit assembly	4.42e-18	3.91e-15
7	ribosomal large subunit assembly	2.47e-05	0.00429
8	RNA decapping	0.00254	0.274
9	oxidative phosphorylation	7.25e-28	4.27e-24
10	sterol metabolic process	5.32e-10	1.66e-07
12	proteasome assembly	3.26e-32	5.77e-28
13	mitochondrial translational initiation	3.15e-05	0.00528
15	tRNA transcription by RNA polymerase III	8.23e-29	7.27e-25

Cluster ID	Term	P-Value	FDR
16	nucleosome disassembly	1.39e-32	3.69e-28
17	polyphosphate metabolic process	1.8e-12	7.95e-10
18	cell wall mannoprotein biosynthetic process	8.46e-12	3.43e-09
19	endonucleolytic cleavage in 5'-ETS of tricistronic rRNA...	8.51e-19	8.06e-16
20	nuclear polyadenylation-dependent ncRNA catabolic proces...	3.26e-28	2.16e-24
22	tRNA aminoacylation for protein translation	1.62e-16	1.2e-13
24	purine ribonucleoside diphosphate catabolic process	4.14e-21	7.09e-18
26	RNA polymerase II preinitiation complex assembly	3.19e-16	2.28e-13
27	ribophagy	0.000533	0.0698
29	isoleucine metabolic process	4.68e-11	1.67e-08
30	vitamin B6 metabolic process	0.000183	0.0263
31	aminophospholipid transport	1.05e-06	0.000216
32	actin cortical patch organization	9.03e-20	9.77e-17
33	termination of RNA polymerase II transcription	2.35e-14	1.34e-11
34	positive regulation of transcription elongation by RNA p...	1.19e-16	9.01e-14
35	mitochondrial transmembrane transport	8.2e-08	1.99e-05
36	nuclear pore localization	5.3e-16	3.65e-13
37	anaphase-promoting complex-dependent catabolic process	5.83e-15	3.55e-12
38	double-strand break repair via break-induced replication	7.03e-17	5.48e-14
39	nuclear-transcribed mRNA catabolic process, RNase MRP-de...	1.33e-16	9.91e-14
40	export across plasma membrane	0.00017	0.0245
41	regulation of SNARE complex assembly	5.3e-16	3.65e-13
42	vesicle tethering involved in exocytosis	1.9e-17	1.55e-14
43	stress granule assembly	7.43e-05	0.0117
44	negative regulation of glycogen metabolic process	1.36e-05	0.00246
45	positive regulation of microtubule polymerization or dep...	1.86e-13	9.65e-11
46	protein import into peroxisome matrix	1.68e-12	7.47e-10
47	negative regulation of rDNA heterochromatin formation	4.84e-14	2.68e-11
48	TOR signaling	7.69e-15	4.63e-12
49	telomere maintenance via recombination	1.2e-09	3.63e-07
50	spliceosomal conformational changes to generate catalyti...	2.08e-25	8.49e-22
53	ribosomal large subunit assembly	9.19e-08	2.22e-05

Supplementary Figure S5. RISK analysis of overrepresented GO BP terms—as defined by Ashburner *et al*. (2000)—in the yeast PPI network (Michaelis *et al*., 2023). RISK identifies 41 GO BP clusters using the Louvain algorithm (Blondel *et al*., 2008), with clusters labeled and positioned near their centroids. A table lists the most significantly overrepresented GO BP term for each cluster, along with cluster IDs, P-values, and FDR corrections.

Yeast Protein–Protein Interaction Network

Michaelis *et al.*, 2023

SAFE with GO BP Clusters (n = 22)

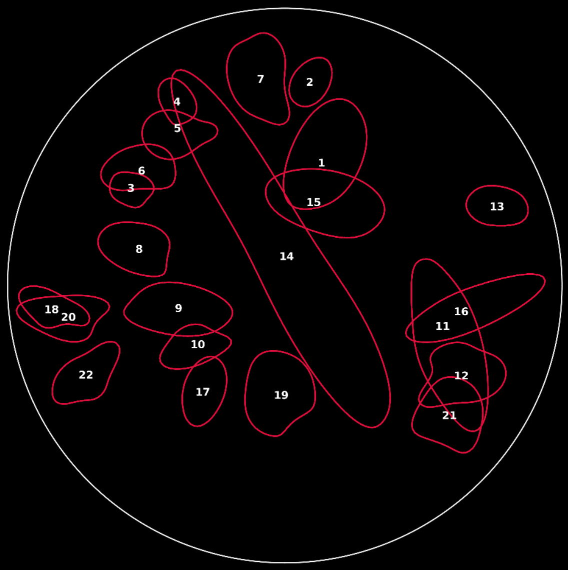

Cluster ID	Term	P-Value	FDR
10	tRNA transcription by RNA polymerase III	3.33e-40	1.58e-36
11	export across plasma membrane	1.14e-08	2.15e-06
12	cell wall (1->3)-beta-D-glucan biosynthetic process	3.85e-07	6.15e-05
13	spliceosomal conformational changes to generate catalyti...	1.78e-35	4.59e-32
14	cytoplasmic translational initiation	5.43e-16	2.23e-13
15	tRNA aminoacylation for protein translation	1.32e-14	4.55e-12
16	Arp2/3 complex-mediated actin nucleation	3.73e-29	8.8e-26
17	proteasome assembly	1.01e-42	5.74e-39
18	anaphase-promoting complex-dependent catabolic process	6.62e-29	1.18e-25
19	regulation of pseudohyphal growth	4.16e-07	6.54e-05
20	nuclear-transcribed mRNA catabolic process, RNase MRP-de...	4.05e-24	4.61e-21
21	Golgi organization	1.04e-17	5.5e-15
22	negative regulation of rDNA heterochromatin formation	4.26e-22	3.67e-19

Cluster ID	Term	P-Value	FDR
1	isoleucine metabolic process	1.52e-16	6.47e-14
2	endonucleolytic cleavage to generate mature 5'-end of SS...	2.93e-46	8.34e-42
3	assembly of large subunit precursor of preribosome	5.48e-16	2.23e-13
4	ribosomal small subunit assembly	1.15e-20	8.39e-18
5	regulation of translational fidelity	0.00238	0.192
6	ribosomal large subunit assembly	1.51e-11	3.78e-09
7	proton motive force-driven ATP synthesis	4.02e-29	8.8e-26
8	nucleosome disassembly	3.78e-37	1.32e-33
9	transcription-coupled nucleotide-excision repair	1.25e-16	5.62e-14

Supplementary Figure S6. SAFE analysis of overrepresented GO BP terms—as defined by Ashburner *et al*. (2000)—in the yeast PPI network (Michaelis *et al*., 2023). SAFE identifies 22 GO BP clusters using the shortest-path algorithm (Baryshnikova, 2016), with clusters labeled and positioned near their centroids. A table lists the most significantly overrepresented GO BP term for each cluster, along with cluster IDs, P-values, and FDR corrections.

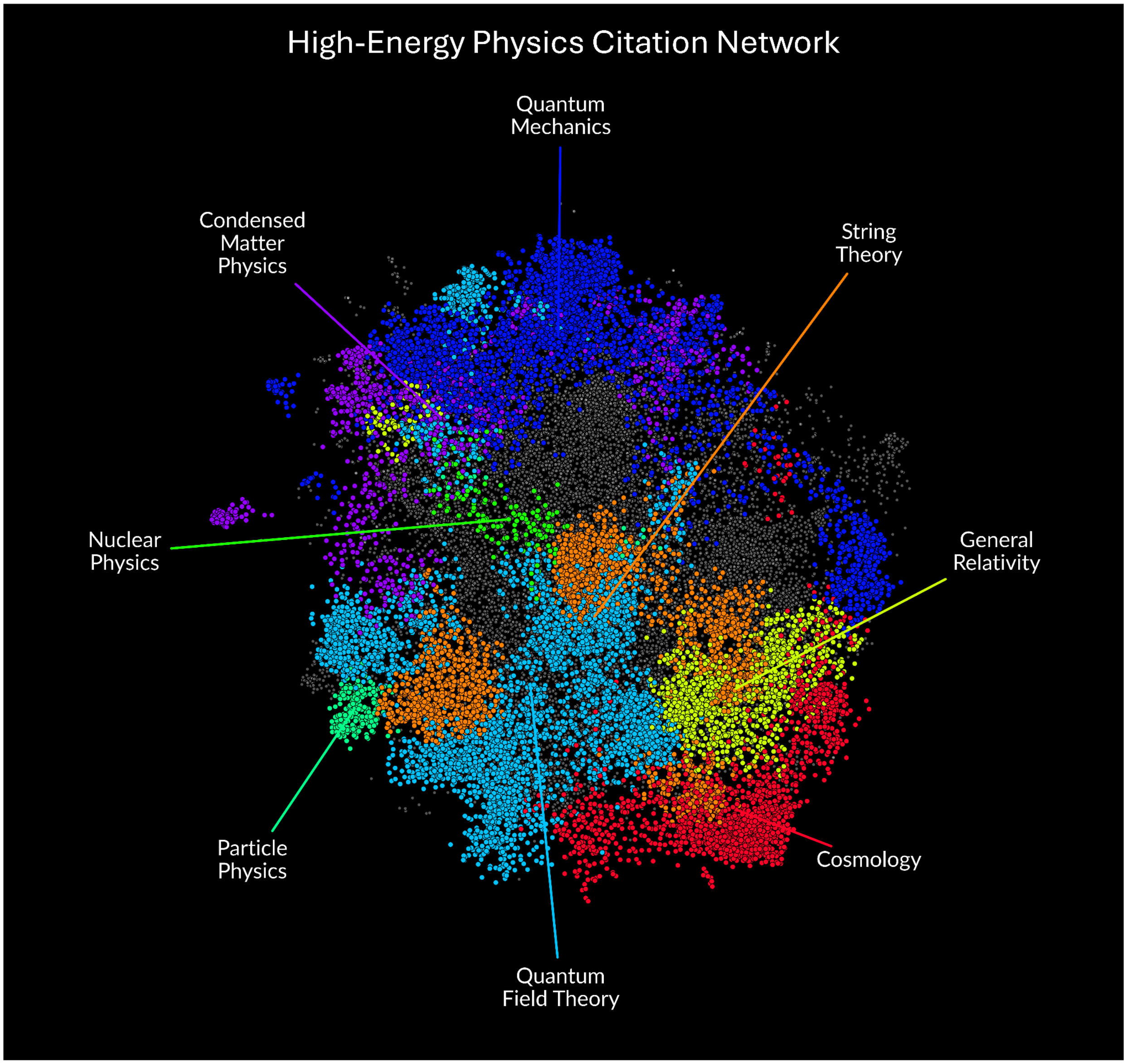

Supplementary Figure S7. RISK analysis of a high-energy physics citation network (20,147 nodes, 331,044 edges; Gehrke *et al.*, 2003; Leskovec *et al.*, 2005; Leskovec and Krevl, 2014). Nodes represent publications, and edges represent citations. The network layout was generated using the ForceAtlas2 algorithm (Jacomy *et al.*, 2014). Using the Leiden algorithm (Traag *et al.*, 2019), RISK identifies 8 distinct clusters representing research subfields ($p < 0.001$, permutation test), including quantum mechanics, particle physics, and cosmology. Thematic overlaps emerge in related subfields, such as general relativity and cosmology.

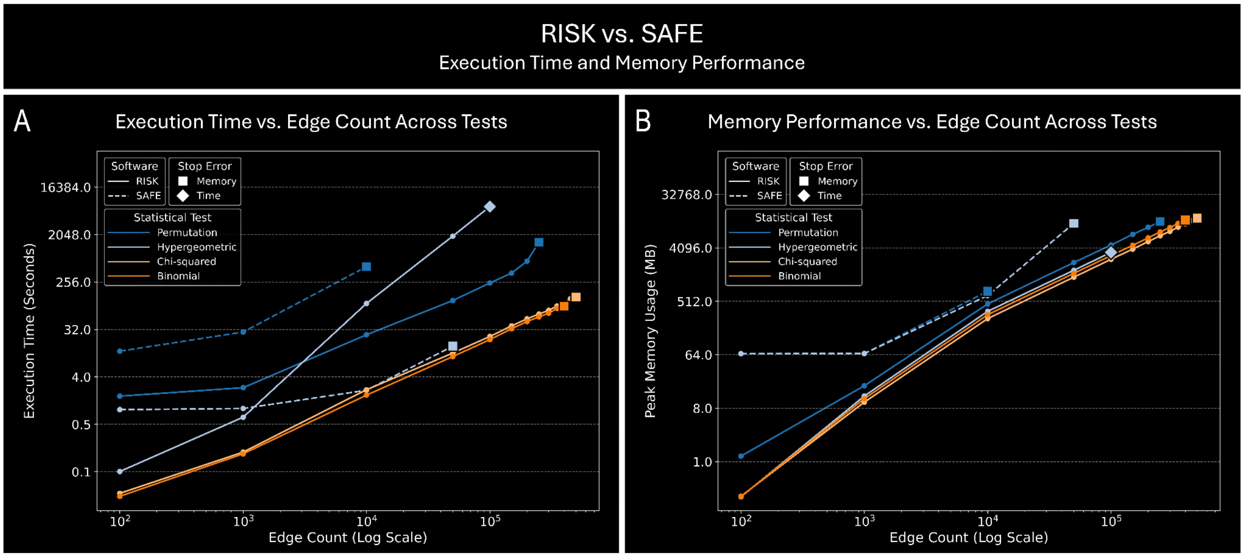

Supplementary Figure S8. Benchmarking execution time and memory usage for RISK and SAFE across multiple statistical tests. Mock scale-free networks (Barabási and Albert, 1999) were generated with twice as many edges as nodes. 1,000 simulated terms were assigned using term-to-gene membership distributions modeled after GO BP terms—as defined by Ashburner *et al*. (2000)—to ensure biologically relevant benchmarking conditions. Benchmarks were performed on a system with an Intel® Core™ i7-4770 CPU (3.40 GHz), 15 GiB RAM, and 928 GB disk space, running Ubuntu 22.04.5 LTS. Each data point represents the average of 5 replicates. Execution time was capped at 6 hours and memory usage at 15 GiB. (**A**) Execution time of RISK and SAFE across 4 statistical tests: permutation, hypergeometric, chi-squared, and binomial. RISK supports all 4 tests, whereas SAFE supports only permutation and hypergeometric. RISK exhibits lower execution times, particularly for computationally intensive workflows such as the permutation test. (**B**) Peak memory usage of RISK and SAFE across the same tests. RISK maintains efficient memory usage and scales robustly across all tests, whereas SAFE shows higher memory demands, especially for the permutation test. RISK successfully processes networks with up to 500,000 edges (250,000 nodes) using the chi-squared test, whereas SAFE fails to process networks beyond 50,000 edges (25,000 nodes) using the hypergeometric test.

References

Ashburner M, Ball CA, Blake JA *et al.* Gene Ontology: Tool for the unification of biology. *Nat Genet* 2000;**25**:25–9.

Barabási A-L, Albert R. Emergence of scaling in random networks. *Science* 1999;**286**:509–12.

Baryshnikova A. Systematic functional annotation and visualization of biological networks. *Cell Syst* 2016;**2**:412–21.

Blondel VD, Guillaume JL, Lambiotte R *et al.* Fast unfolding of communities in large networks. *J Stat Mech* 2008;**P10008**.

Costanzo M, VanderSluis B, Koch EN *et al.* A global genetic interaction network maps a wiring diagram of cellular function. *Science* 2016;**353**.

Gehrke J, Ginsparg P, Kleinberg JM. Overview of the 2003 KDD Cup. *SIGKDD Explor* 2003;**5**:149–51.

Jacomy M, Venturini T, Heymann S, *et al*. ForceAtlas2, a continuous graph layout algorithm for handy network visualization designed for the Gephi software. *PLoS ONE* 2014;**9**:e98679.

Leskovec J, Kleinberg J, Faloutsos C. Graphs over time: Densification laws, shrinking diameters and possible explanations. *ACM SIGKDD Int Conf Knowl Discov Data Min* 2005;:177–87.

Leskovec J, Krevl A. SNAP Datasets: Stanford Large Network Dataset Collection. 2014;.

Michaelis AC, Brunner AD, Zwiebel M, Meier F, Strauss MT, Bludau I, Mann M. The social and structural architecture of the yeast protein interactome. *Nature* 2023;**624**:192–200.

Traag VA, Waltman L, van Eck NJ. From Louvain to Leiden: Guaranteeing well-connected communities. *Sci Rep* 2019;**9**:5233.
